# Link Prediction with Continuous-Time Classical and Quantum Walks

**DOI:** 10.3390/e25050730

**Published:** 2023-04-28

**Authors:** Mark Goldsmith, Harto Saarinen, Guillermo García-Pérez, Joonas Malmi, Matteo A. C. Rossi, Sabrina Maniscalco

**Affiliations:** 1 Algorithmiq Ltd., Kanavakatu 3 C, FI-00160 Helsinki, Finland; 2 Complex Systems Research Group, Department of Mathematics and Statistics, University of Turku, FI-20014 Turku, Finland; 3 QTF Centre of Excellence, Department of Physics, Faculty of Science, University of Helsinki, FI-00014 Helsinki, Finland; 4 InstituteQ-The Finnish Quantum Institute, University of Helsinki, FI-00014 Helsinki, Finland; 5 QTF Centre of Excellence, Department of Applied Physics, Aalto University, FI-00076 Aalto, Finland; 6 InstituteQ-The Finnish Quantum Institute, Aalto University, FI-00076 Aalto, Finland

**Keywords:** link prediction, protein–protein interaction networks, random walks, quantum walks

## Abstract

Protein–protein interaction (PPI) networks consist of the physical and/or functional interactions between the proteins of an organism, and they form the basis for the field of network medicine. Since the biophysical and high-throughput methods used to form PPI networks are expensive, time-consuming, and often contain inaccuracies, the resulting networks are usually incomplete. In order to infer missing interactions in these networks, we propose a novel class of link prediction methods based on continuous-time classical and quantum walks. In the case of quantum walks, we examine the usage of both the network adjacency and Laplacian matrices for specifying the walk dynamics. We define a score function based on the corresponding transition probabilities and perform tests on six real-world PPI datasets. Our results show that continuous-time classical random walks and quantum walks using the network adjacency matrix can successfully predict missing protein–protein interactions, with performance rivalling the state-of-the-art.

## 1. Introduction

The link prediction problem has long been an active area of research, with applications ranging from friendship recommendation in social networks [1,2,3] to finding missing interactions between proteins [4,5]. In this paper, we were interested in the latter. For general surveys in link prediction, we refer to [6,7,8].

One particularly successful class of link prediction methods is those based on random walks [5,9,10]. Random walk algorithms have been explored more generally throughout the field of network science, and many different applications exist. These include the ranking of web pages using PageRank [11,12], collaborative filtering [13], and computer vision [14]. Many random walk link prediction algorithms have also been studied [5,15]. These methods typically rely on discrete-time random walks.

In contrast, in this paper, we propose a class of link prediction methods based on continuous-time random walks. Moreover, the continuous-time setting allowed us to propose a new link prediction method using quantum walks, which closely resembles the classical method described here.

Continuous-time quantum walks, initially proposed in [16], are the quantum analogues of continuous-time classical random walks, which describe the propagation of a particle over a discrete set of positions. Together with their discrete-time counterpart [17], they have received much attention for their applications in quantum information processing [18,19], quantum computation [20], and quantum transport [21]. However, only a few recent methods have attempted to use quantum walks for link prediction, using their discrete-time [22] and continuous-time [23] variations. While the methods described here are *quantum-inspired*, since they were implemented classically, we can foresee that these will be even more efficient if run on quantum devices. Continuous-time quantum walks have already been implemented on various physical platforms [24], including optical setups [25,26,27,28,29] and superconducting devices [30,31], and they can also be simulated on gate-based quantum computers [32,33].

In order to evaluate our proposed methods, we conducted experiments on several networks and found that both the classical and quantum walks outlined here are particularly good at finding missing links in protein–protein interaction (PPI) networks. Protein–protein interactions play a critical role in all cellular processes, ranging from cellular division to apoptosis. Elucidating and analysing PPIs is thus essential to understand the underlying mechanisms in biology and, eventually, to unveil the molecular roots of human disease [34]. Indeed, this has been a major focus of research in recent years, providing a wealth of experimental data about protein associations [35,36]. Current PPI networks, called interactomes, have been constructed using a number of techniques, but despite the enormous advancement, the current coverage of PPIs is still rather poor (for example, it is estimated that only around 10% of interactions in humans are currently known [37]). Additionally, despite considerable improvements in high-throughput (HTP) techniques, they are still prone to spurious errors and systematic biases, yielding a significant number of false positives and false negatives. This limitation impedes our ability to assess the true quality and coverage of the interactome.

Recently, a number of algorithms have been developed to predict protein–protein interactions. In a recent study by Kovács et al. [4] (see also [38,39]), a novel PPI-specific link predictor was proposed. Their link predictor was biologically motivated by the so-called L3 principle, and it was shown to be superior to other general link predictors when applied to PPI data. The exceptional success of the L3 framework is rooted in its ability to capture the structural and evolutionary principles that drive PPIs. The results of Kovács and collaborators proved that, contrary to the current network paradigm, interacting proteins are not necessarily similar and similar proteins do not necessarily interact, questioning the traditional validation strategy based on the biological similarity of the predicted protein pairs.

However, the L3 link prediction method, considered the most-successful to date for PPIs, as well as most other existing link prediction methods are not without limitations. The most-common approaches cannot find interactions for self-interacting proteins or links between proteins that have long shortest paths between them. Given the low coverage of the current PPI databases, this can be a significant drawback. It is, therefore, highly desirable to complement the existing frameworks with methods relying on the exploration of the whole network, and consequently be able to predict edges whose corresponding nodes may be far away in the network. Thus, we propose novel quantum- and classical-random-walk-based link prediction methods that can potentially traverse the entire network and simultaneously predict self-edges.

## 2. Materials and Methods

Consider a network modelled by an undirected and unweighted graph G=(V,E), where *V* is the set of nodes of size *n* and *E* is the set of edges. We allowed for the existence of self-edges, so that for any node *i*, the edge (i,i) may or may not be present in *E*. The *adjacency matrix* of *G* is the n×n matrix defined by

$$A=\left(A_{ij}\right)=\left\{\begin{array}{cc} 1, & \;\mathrm{if}\;(i,j)\in E,\\ 0, & \;\mathrm{if}\;(i,j)\notin E. \end{array}\right.$$

The *graph Laplacian* is defined as L=D−A, where *D* is the *degree matrix* defined by $D=\mathrm{diag}\left(\sum_{j}A_{1j},\ldots ,\sum_{j}A_{nj}\right)$.

The link prediction problem is to infer missing links in a network *G*, using only the information provided by the structure of *G*. Thus, a link prediction algorithm typically gives a ranking of all the non-edges (pairs of nodes that are not directly connected in *G*) based on some proposed scoring scheme.

We now present a rather general scoring scheme for ranking the non-edges of a graph based on state transition probabilities resulting from quantum and classical random walks; the precise details of the walks we employed are described in the next subsections. For now, it suffices to consider the notion of a probability transition matrix that evolves over time, denoted by P(t); for a graph *G*, the probability of the walker being at node *v* at time *t*, given that it began at node *u*, is thus $P_{uv}\left(t\right)$. For a fixed time *t*, we define the score S(i,j;t) between two non-adjacent nodes *i* and *j* at time *t* to be

$$S(i,j;t)=\left\{ \begin{array}{cc} P_{ij}(t)\left(k_{i}+k_{j}\right)\;i\neq j & \left(1\right)\\ \frac{1}{2}\sum_{u\in N(i)}P_{iu}(t)\;i=j, & \left(2\right) \end{array}\right.$$

where *N*(*v*) denotes the set of nodes adjacent to *v* (possibly including *v* itself) and $k_{v}=\sum_{j}A_{vj}$ is the degree of node v. Equations (1) and (2) handle the cases of distinct nodes and self-edges, respectively. The scoring scheme in Equation (1) is based on the intuition that two nodes *i* and *j* should likely be connected if the walk is more likely to move from *i* to *j* than to other nodes. We also scale these probabilities by the node degrees so that high-degree nodes have a higher preference, similar to the preferential attachment link prediction method [40,41]. Further, Equation (2) claims that the properties of the walker in the neighbourhood of the node determines the likelihood of a self-edge. While the score in Equation (1) is superficially similar to the one proposed in [5], the fact that we use continuous-time walks leads to several key differences: the continuous-time nature of our method allows for a wider range of time parameters *t* to use; in the continuous-time setting, there is symmetry in the transition probabilities, i.e., $P_{ij}\left(t\right)=P_{ji}\left(t\right)$ for all nodes i,j; finally, there is a close relationship in the implementation of classical and quantum walks in the continuous-time setting.

Regardless of which type of walk is used, we must choose a value *t*, representing the time duration of the walk. We start the walk at time $t_{0}=0$ and let it run for a time *t*, at which point we extract the scores for the target edges from the probability distributions. In the case of a continuous-time classical random walk, the expected time it takes for a random walker to leave a node *i* is $1/k_{i}.$ This motivates the idea that the amount of time we let the walk run should be related to the degree distribution of the network. In our experiments, we tested a few small multiples of the value 1/〈k〉, where 〈k〉 is the average node degree in the graph, and report the value yielding the best results (see the results in Section 3).

### 2.1. Continuous-Time Random Walks

A continuous-time (classical) random walk (CRW) is a Markov process with state space *V* characterised by an initial distribution p(0) over the set of nodes and a rate matrix *Q* that has null row sum $\sum_{k}Q_{jk}=0$ for all *j*. Here, we considered edge-based random walks [42] (as opposed to node-based), which are characterised by setting Q=−L, where *L* is the Laplacian of the underlying graph. In this case, the evolution of the probability vector p(t) is governed by the equation:

(3) p(t)=p(0)P(t),

where $P\left(t\right)=e^{-tL}$ is the probability transition matrix, which has the elements $P_{ij}\left(t\right)=\langle j|e^{-itL}|i\rangle$, where *i* and *j* are standard basis vectors.

Intuitively, the random walker operates as follows. Every edge of the graph is associated with an independent Poisson process with unit intensity. When the walker is at some node, it will remain there until one of the Poisson processes at an incident edge jumps, at which point, the walker follows that edge to the corresponding neighbour, and the process repeats. Note that this implies that, on average, a random walker will spend less time waiting at a higher-degree node than at a lower-degree node. Furthermore, this method will assign non-zero probabilities to all pairs of nodes in a connected component, due to the continuous-time nature of the walk.

### 2.2. Continuous-Time Quantum Walks

In contrast to a classical random walk, a quantum walk on a network evolves according to the laws of quantum physics. A major implication of this is that the trajectories of the walker across the network can interfere constructively or destructively. This interference causes the evolution of the quantum walker to sometimes be significantly different from the classical one [17,43].

A continuous-time quantum walk (QW) [16] on a graph *G* is defined by considering the Hilbert space H spanned by the orthonormal vectors ${\left\{|i\rangle \right\}}_{i=1}^{n}$, corresponding to the *n* nodes of the graph and the unitary transformation U(t). This transformation implies that the state vector in H at a time *t* after starting from initial time $t_{0}=0$ is given by the evolution:

(4) $$|\psi (t)\rangle =U\left(t\right)|\psi (0)\rangle ,$$

where $U\left(t\right)=e^{-itH}$ is the unitary evolution operator and *H* is the Hamiltonian. In general, the Hamiltonian *H* can be almost any Hermitian matrix related to *G* as long as it describes the structure of the network [19], but the most-common choices are the graph adjacency matrix *A* or the Laplacian *L* [44]. We also note that, in the classical random walk, the rate matrix *Q* is required to have a null row sum so that it is probability-conserving, and thus, the Laplacian *L* is a valid choice. However, for quantum walks, no such restriction exists, and a wider range of walks can be considered by modifying the Hamiltonian, as long as it remains Hermitian [45]. For example, the graph adjacency matrix can be used as a Hamiltonian, but not as a classical rate matrix since its rows do not sum to zero. In this paper, we used both the adjacency and Laplacian matrices as the Hamiltonians separately and, therefore, can compare different realisations of quantum walks for the link prediction task.

In order to obtain a probability transition matrix analogous to the one in Equation (3), we must take the square of the modulus of the entries of U(t). The entries of the probability transition matrix are given by

(5) $$P_{ij}\left(t\right)=\left|\langle j\right|e^{-itH}{\left|i\rangle \right|}^{2}.$$

These transition probabilities can then be used to compute scores for non-edges as described in Equations (1) and (2) above. Note that, contrary to the classical case, where randomness comes from stochastic transitions between states, in the quantum walk, the state transitions are deterministically governed by the Schrödinger equation, and the randomness results from the measurement and collapse of the wave function.

Our motivation for the usage of continuous- rather than discrete-time walks is threefold: there is a close resemblance between the classical and quantum versions via the matrix exponential, which allows both methods to be easily compared; having a real, rather than an integer-valued hyperparameter *t* allows for a wider range of results to be explored and also permits non-zero scores to be assigned to all pairs of non-neighbouring nodes within a connected component. We emphasise that the usage of continuous-time quantum walks for link prediction is a new direction of research, with very few studies conducted so far. The method proposed in [23], in particular, appears to be competitive with some state-of-the-art link prediction methods in certain real networks. While some aspects of their algorithm are similar to the quantum version of our algorithm, the implementation details and calculation of the link prediction scores are very different. Moreover, their algorithm requires entanglement with an additional ancilla. While this would be feasible in a hypothetical implementation on a quantum computer, the typical sizes of relevant real networks are far beyond the capabilities of current and near-term quantum hardware. Simulations on classical computers are required, but the presence of the extra ancilla increases the complexity of the simulations.

### 2.3. Datasets and Metrics

We tested our link prediction methods on six different PPI networks. Four networks were *Homo sapiens* (human) PPI networks: we used the physical, multi-validated interactions from v4.4.219 of BioGRID [46], the high-quality binary and co-complex interactions from the HINT database [47], the interactions proven by 2 or more pieces of experimental evidence from APID [48,49] (downloaded on 1 March 2023), and the experimentally validated interactions from the Integrated Interactions Database (IID) [50], Version 2021-05. Furthermore, we also tested our methods on the interactions of the organism *Saccharomyces cerevisiae* (yeast) from BioGRID and HINT just described.

**Figure 1:**
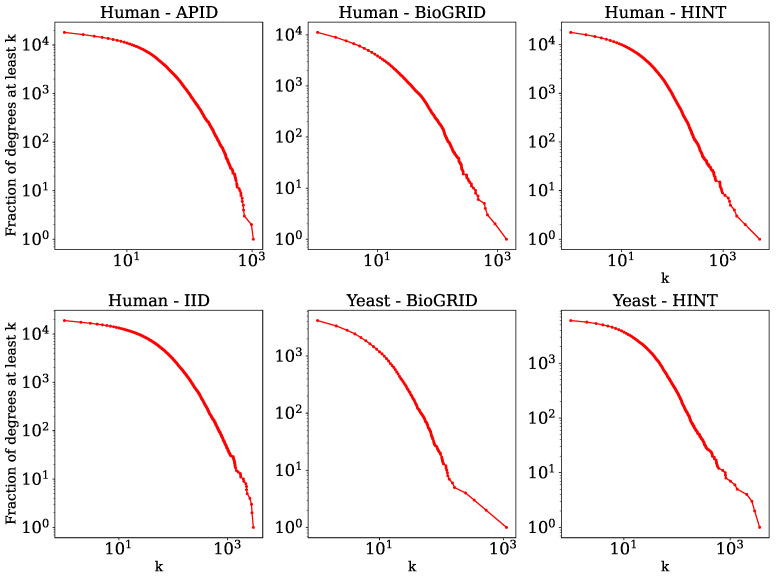
Complementary cumulative degree distributions. For each degree value *k* (*x*-axis), the proportion of nodes with degree greater than or equal to *k* (*y*-axis) is shown, each on a logarithmic scale.

Some statistics of these networks are listed below in Table 1, and their degree distributions are shown in Figure 1. We observed from these statistics that the networks have high clustering and that they are very sparse. Furthermore, the networks are approximately scale-free [51], which is typical of biological networks. One distinguishing feature of PPI networks compared to most other complex networks is that they contain self-edges, which represent the ability of a protein to interact with itself.

Since the ground truth of the considered PPI networks is of course unknown, we proceeded to test the algorithms using cross-validation. For each dataset, we randomly removed P% of the edges in the original network, for P∈{10,20,30,40,50}, and reserved these edges as positive test cases. All of the non-edges (including self-edges that are not present in the network) were used as negative testing data. These positive and negative edges were used to evaluate the methods, and the remaining (100−P)% existing edges were used for running the models in question. In other words, after removing the P% of the edges, the non-edges were ranked by sorting them in descending order according to their scores, and the edges with higher scores were deemed most likely to exist. This ranking was then compared to the evaluation set to see how well the positive test cases were ranked. This process was repeated 10 times for each *P*, and the results of the accuracy metrics were averaged (see the results in Section 3).

In order to compare the rankings of the edges of the methods under consideration, we used the areas under the precision–recall and receiver operator characteristic curves, two metrics that are typically used in link prediction and other binary classification problems. Hence, we define

$$\begin{array}{c} \mathrm{true}\;\mathrm{positive}\;\mathrm{rate}\;=\;\mathrm{recall}=\frac{\mathrm{TP}}{\mathrm{TP}+\mathrm{FN}},\\ \mathrm{precision}=\frac{\mathrm{TP}}{\mathrm{TP}+\mathrm{FP}},\\ \mathrm{false}\;\mathrm{positive}\;\mathrm{rate}=\frac{\mathrm{FP}}{\mathrm{FP}+\mathrm{TN}}, \end{array}$$

where TP = true positive, FP = false positive, FN = false negative, and TN = true negative. In order to calculate each of these from the rankings, a threshold that serves as a cut-off rule has to be selected (the predictions above the thresholds are classified as positive and below it as negative). Our two metrics were calculated by varying this threshold trough the rankings. Firstly, we considered the area under the precision–recall curve (AuPR). Precision–recall curves plot the recall on the *x*-axis against precision on the *y*-axis. In order to reduce this curve to a single number, the area under the curve is used, and this also circumvents the problem of choosing an arbitrary score threshold at which to distinguish predicted positives from negatives. Note that the AuPR focuses only on performance relative to the positive class, an important consideration when the ratio of positive cases to negatives cases is small, as is the case in most networks and especially in PPI networks (these networks are extremely sparse; see Table 1). As a secondary metric, we considered the area under the receiver operating characteristic curve (AuROC) [52], which plots the false positive rate versus the recall. It can be interpreted as the probability that the classifier will rank a positive case, chosen uniformly at random from the positive set, higher than a negative one, chosen uniformly at random from the negative set [53]. Thus, a random classifier has an AuROC equal to half and a perfect classifier has an AuROC equal to one. We emphasise that the AuPR is widely accepted as the preferred metric for link prediction, due to the large class imbalance mentioned above [54,55].

## 3. Results

In order to test our methods, we selected five other popular link prediction methods to compare against: *L3* relies on a weighted counting of paths of length three and was designed specifically to predict links in PPI networks [4]; *preferential attachment* (PA) defines a score between two disconnected nodes by multiplying their degrees [40,41]; *common neighbours* (CN) is a straightforward heuristic that assigns a score to the node pair (u,v) defined by the number of neighbours that *u* and *v* have in common; *Adamic-Adar* (AA) is an adaptation of the common neighbours idea, but adds more weight to less-connected neighbours [1]; the *structural perturbation method* (SPM) uses perturbations of the adjacency matrix of a graph in order to estimate its predictability [56]. While the SPM has shown great success as a general link prediction method [6,57], it is yet to be tested extensively on PPI networks. For the SPM, we used $p^{H}=0.1$ and averaged the results over 10 runs, as was performed in the original paper [56].

The following tables show the average AuPR and AuROC values for the six different networks described in Section 2.3. Each value was averaged over 10 runs (10 randomly selected edge removals), and the highest value for each network is shown in bold. We compared three variations of our proposed methods, labelled as “QW-A”, “QW-L”, and “CRW”, referring to quantum walks using the network adjacency matrix as the Hamiltonian, quantum walks using the network Laplacian matrix as the Hamiltonian, and classical random walks, respectively.

**Figure 2:**
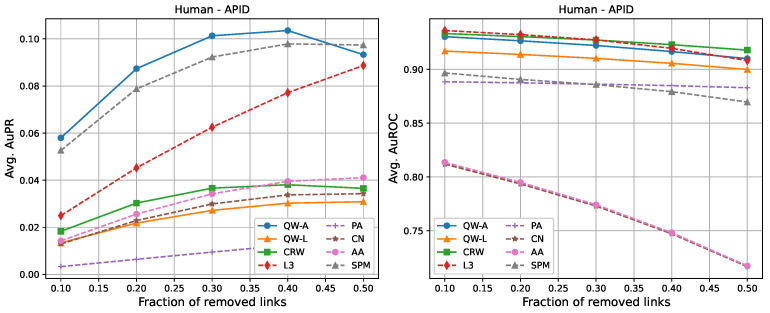
Average areas under the precision–recall curve (**left**) and average areas under the receiver operating characteristic curve (**right**) as a function of the fraction of true links that were removed from the APID *Homo sapiens* PPI network [48,49]. Plotted values are the averages over 10 runs. Our walks used a hyperparameter of t=3/〈k〉.

**Figure 3:**
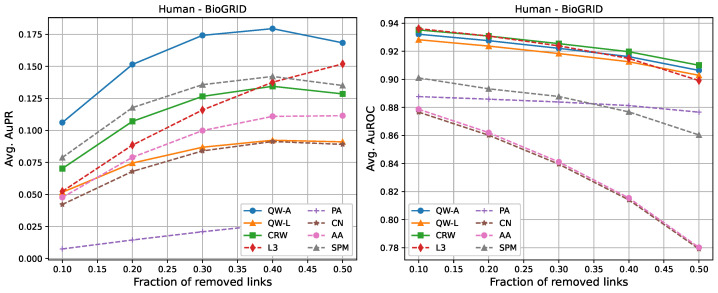
Average areas under the precision–recall curve (**left**) and average areas under the receiver operating characteristic curve (**right**) as a function of the fraction of true links that were removed from the BioGRID *Homo sapiens* PPI network [46]. Plotted values are the averages over 10 runs. Our walks used a hyperparameter of t=2/〈k〉.

**Figure 4:**
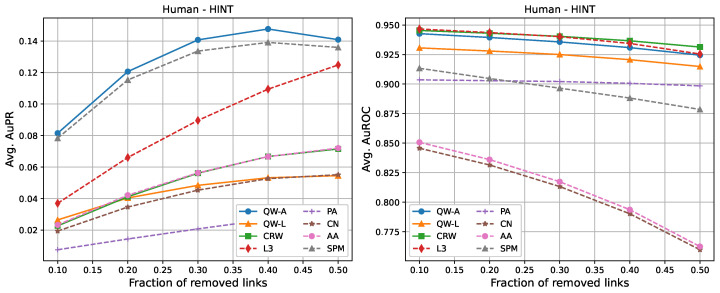
Average areas under the precision–recall curve (**left**) and average areas under the receiver operating characteristic curve (**right**) as a function of the fraction of true links that were removed from the HINT *Homo sapiens* PPI network [47]. Plotted values are the averages over 10 runs. Our walks used a hyperparameter of t=3/〈k〉.

**Figure 5:**
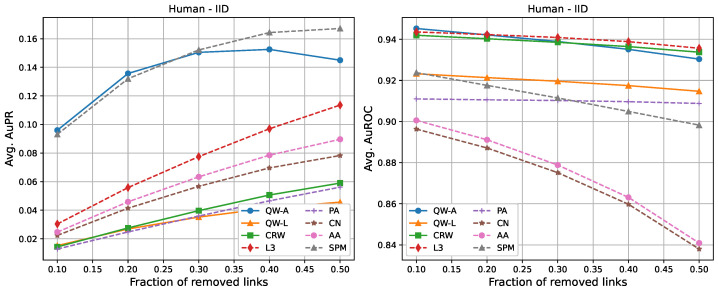
Average areas under the precision–recall curve (**left**) and average areas under the receiver operating characteristic curve (**right**) as a function of the fraction of true links that were removed from the IID *Homo sapiens* PPI network [50]. Plotted values are the averages over 10 runs. Our walks used a hyperparameter of t=4/〈k〉.

**Figure 6:**
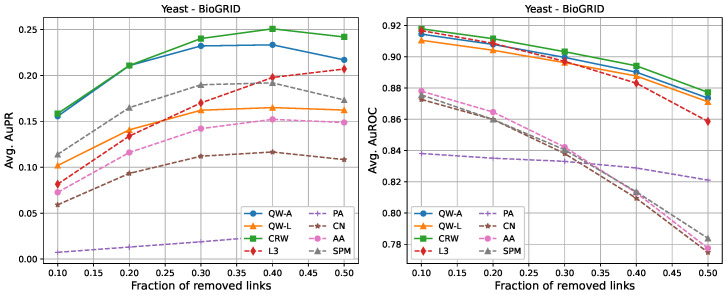
Average areas under the precision–recall curve (**left**) and average areas under the receiver operating characteristic curve (**right**) as a function of the fraction of true links that were removed from the BioGRID *Saccharomyces cerevisiae* PPI network [46]. Plotted values are the averages over 10 runs. Our walks used a hyperparameter of t=2/〈k〉.

**Figure 7:**
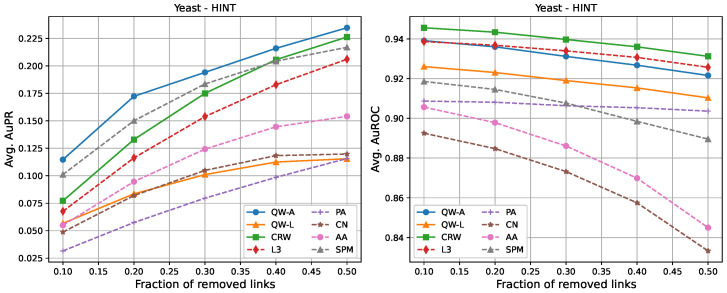
Average areas under the precision–recall curve (**left**) and average area under the receiver operating characteristic curve (**right**) as a function of the fraction of true links that were removed from the HINT *Saccharomyces cerevisiae* PPI network [47]. Plotted values are the averages over 10 runs. Our walks used a hyperparameter of t=2/〈k〉.

For completeness, in Figure 2, Figure 3, Figure 4, Figure 5, Figure 6 and Figure 7, we also include plots showing the relationship of the area under the precision–recall curve and area under the ROC curve as a function of the edge removal fraction.

In terms of area under the precision–recall curve (AuPR), the quantum walk with the adjacency Hamiltonian (QW-A) showed the best results overall. When 10% of the edges were removed, the QW-A had a higher average AuPR than all other benchmarked methods. This also held when 50% of the edges were removed, except in three cases. For the secondary metric, AuROC, the three best methods appeared to be QW, CRW, and L3; while L3 had the highest AuROC in half of the networks at the 10% removal level by a small margin, CRW had the highest AuROC at the 50% level in all but one network.

## 4. Discussion

**Figure 8:**
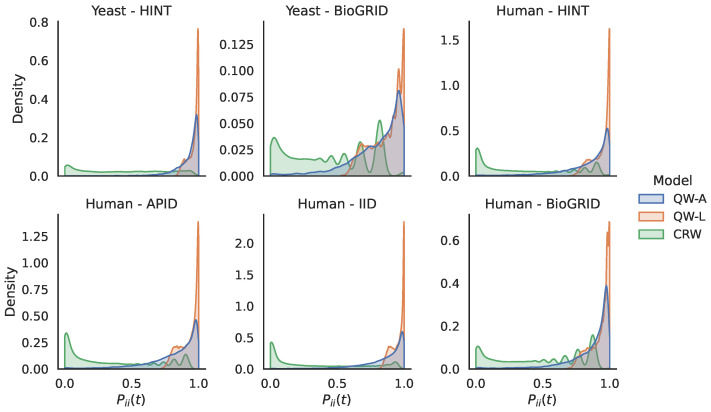
Comparison of return probabilities for the quantum and classical random walk methods on the 6 networks studied. For each network, we show kernel density estimations of the return probabilities $P_{ii}\left(t\right)$, for every node *i*. The values of *t* used are those for which the AuPRs and AuROCs were presented above.

**Figure 9:**
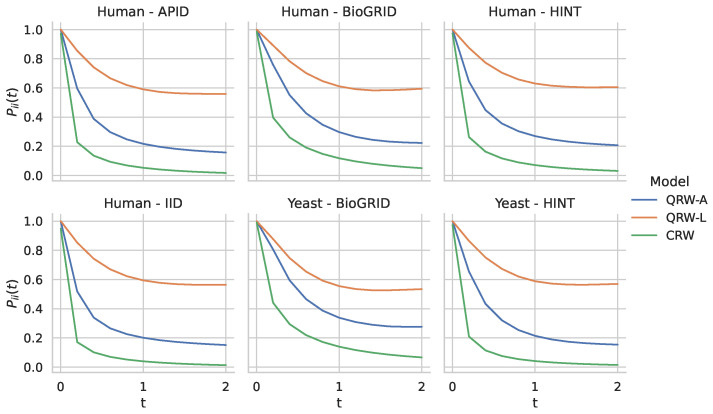
Comparison of return probabilities for the different quantum and classical random walk methods on the 6 networks studied. For each network, we show the the average value of $P_{ii}\left(t\right)$, averaged over all nodes, for values of *t* in the range (0,2).

The experimental results in the previous section showed that our methods performed well on a variety of PPI networks. In particular, we saw that our quantum walk with the adjacency Hamiltonian method yielded the best overall performance of all algorithms tested with respect to the area under the precision–recall curve. Furthermore, the adjacency Hamiltonian always beat the Laplacian as the better choice when comparing the results of quantum walks. One possible explanation for this is that the inclusion of node degrees on the diagonal of the Hamiltonian for the Laplacian matrix caused walkers to remain at nodes for longer periods of time, thus preventing them from adequately exploring the rest of the network. In order to explore this further, in Figure 8, we show the distribution of the return probabilities $P_{ii}\left(t\right)$ over all nodes *i* for the various networks studied. Indeed, we see that the QW-L had a large spike close to 1.0 for all of the networks, indicating that the majority of nodes were never departed from when using the Laplacian Hamiltonian. In order to verify that this claim holds for other values of *t*, in Figure 9, we compare the return probabilities, averaged over all nodes, for various values of t. We see that the QW-L always had the largest average return probability, while the QW-A had an average return probability that was less than the QW-L, but larger than the CRW.

Comparing the QW-A to the CRW, Table 2 and Table 3 above show that the former had a higher area under the precision–recall curve for all networks, except the Yeast-BioGRID network. One interesting property of this network is that it has the highest proportion of self-edges (826 self-interacting proteins out of 4186 proteins; see Table 1) of all the networks considered. In order to test the hypothesis that the CRW performs better when the proportion of self-edges is high, we repeated our experiments on the Yeast-BioGRID network, but this time did not use any self-edges for scoring. We found that the change in AuPR was negligible and that the CRW still had a slightly higher AuPR than the QW-A. Therefore, we do not believe that the high proportion of self-edges plays a significant role in explaining the better performance of the CRW for this network.

**Figure 10:**
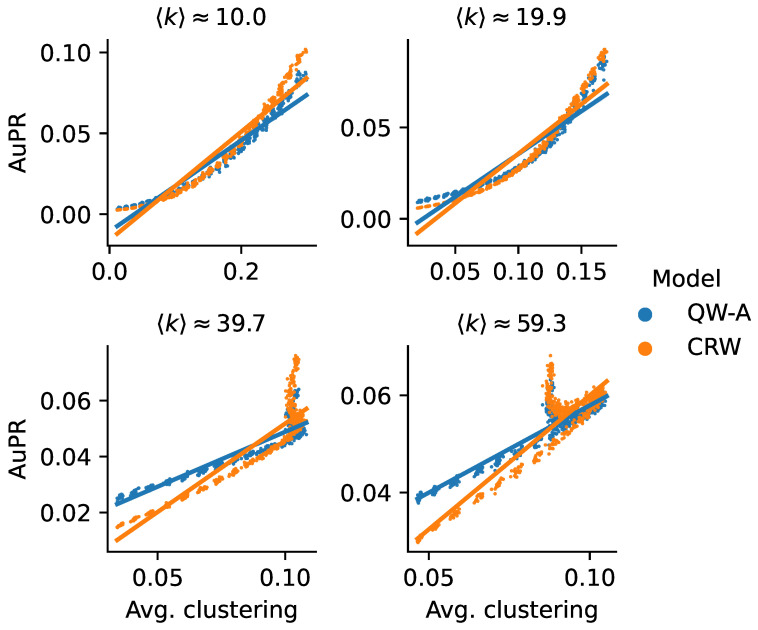
AuPRs for scale-free networks with tunable clusterings. Four different settings are shown, corresponding to different (approximate) average degrees. In each setting, the average clustering was varied to produce different networks. In the resulting networks, half of the edges were reserved for testing, and the remaining network was used to run the QW-A and CRW link prediction methods on. Each point corresponds to the AuPR of a generated network; solid lines show linear fits. Each plot title shows the average degree 〈k〉, averaged over all networks.

Another possible explanation for the higher AuPR of the CRW on the Yeast-BioGRID network may be due to its relatively high clustering compared to the other networks. In order to test this hypothesis, we used a theoretical model to generate scale-free networks with tunable average clusterings [58]. Using this model, we generated scale-free networks with a variety of average clusterings while holding the average degree constant, up to minor random fluctuations. We then used these networks to run the QW-A and CRW using the same cross-validation method described above, with half the edges being removed for testing, in order to compare their performance. In Figure 10, we see that, in all four cases, there was indeed a trend confirming that the QW-A has a better performance when clustering is low, while CRW performs better when clustering is higher. While these theoretically generated networks may not be accurate models of true PPI networks, the effect of clustering on classical and quantum walks remains an interesting topic for future research.

Finally, we mention a few points about the computational complexity of our algorithm and its implementation. The bottleneck of our algorithm, in either the classical or quantum case, is the computation of the matrix exponential appearing in Equations (3) and (5), which is a very well-studied problem with a long history [59]. Our experiments were performed using the “matrix_exp” function in PyTorch [60], which is an implementation of the Taylor polynomial approximation algorithm described in [61]. The problem was thus reduced to a constant number of matrix multiplications, another well-studied problem that can be solved more quickly than the naive $O(n^{3})$ method, for example with Strassen’s algorithm or its variations [62]. It is also worth noting that, in this implementation of matrix exponentiation, and many others, the norm of the matrix being exponentiated has an impact on running time, so that using a small *t*, as tends to be the case in our algorithm, may help in this regard.

In order to compare the running times of the link prediction methods studied here, each method was implemented in python 3.10 and vectorised where possible. The methods were then run on the six networks described in Section 2.3, without removing any edges. The experiments were carried out on a setup consisting of 16 cores and 112 GB of RAM. The results of the running times are shown in Table 4. In general, L3, PA, CN, and AA had the fastest running times, but had low AuPR when compared to the QW-A and SPM (Figure 2, Figure 3, Figure 4, Figure 5, Figure 6 and Figure 7, left). Of the general link prediction methods, the SPM typically had the highest AuPR, but it is computationally demanding due to the need to calculate the eigenvectors and eigenvalues of the perturbed adjacency matrix many times. The QW-A is indeed the most-promising of the methods considered, since its runtime was several times faster than the SPM, while outperforming the SPM in every case, except two, in which case, the QW-A had the higher AuROC (Figure 2 and Figure 5 and Table 5 and Table 6).

## 5. Conclusions

Although experimental methods have greatly improved in the past ten years, most interactomes remain far from being complete. It is, therefore, important to discover new computational methods for inferring interactions from incomplete datasets. We described a class of algorithms based on continuous-time walks that rank among the best link prediction methods tested on PPI networks.

Furthermore, the continuous-time quantum walks described here are among the first successful quantum-inspired link prediction methods. Although we found that using the reciprocal of the average degree provided a good time length for which to run the walks, many further options can still be explored: using cross-validation to choose a more optimal value or using times that depend on the walker’s location are immediate candidates. Another open direction of research involves the choice of the Hamiltonian. Our experimental results demonstrated a strong sensitivity on the Hamiltonian used for controlling the quantum walks. While the adjacency matrix yielded better results than the Laplacian on the networks we tested, it would be beneficial to understand why this is the case. This also indicates the potential for improvement if better Hamiltonians can be found for the purpose of link prediction. Further investigations in this direction may yield better methods and insights into both networks being studied and the quantum walks being employed.

## Figures and Tables

**Table 1:** Some properties of the networks that were tested. |V|: number of nodes, |E|: number of edges, 〈k〉: average degree, ρ: network density, C: average clustering, A: assortativity, SIPs: number of self-interacting proteins (self-edges).

Network	|V|	|E|	〈k〉	ρ	*C*	*A*	SIPs
Yeast-BioGRID	4186	20,053	9.581	0.002	0.306	−0.080	826
Yeast-HINT	6025	92,201	30.606	0.005	0.304	−0.129	1837
Human-BioGRID	11,134	79,536	14.287	0.001	0.200	−0.063	1254
Human-HINT	17,818	256,972	28.844	0.002	0.129	−0.059	5223
Human-APID	18,173	265,216	29.188	0.002	0.086	−0.082	2488
Human-IID	18,925	560,628	59.247	0.003	0.126	−0.085	4684

**Table 2:** Area under the precision–recall curve for 10% edge removals, averaged over 10 runs. The highest AuPR for each network is shown in bold.

AuPR: 10% Removal								
**Network**	**QW-A**	**QW-L**	**CRW**	**L3**	**PA**	**CN**	**AA**	**SPM**
Human-APID	**0.058**	0.013	0.018	0.025	0.003	0.013	0.014	0.053
Human-BioGRID	**0.106**	0.052	0.070	0.052	0.007	0.042	0.048	0.079
Human-HINT	**0.081**	0.026	0.023	0.037	0.008	0.019	0.023	0.078
Human-IID	**0.096**	0.015	0.014	0.030	0.013	0.022	0.025	0.093
Yeast-BioGRID	0.156	0.102	**0.158**	0.082	0.007	0.059	0.073	0.114
Yeast-HINT	**0.115**	0.057	0.077	0.068	0.032	0.049	0.055	0.101

**Table 3:** Area under the precision–recall curve for 50% edge removals, averaged over 10 runs. The highest AuPR for each network is shown in bold.

AuPR: 50% Removal								
**Network**	**QW-A**	**QW-L**	**CRW**	**L3**	**PA**	**CN**	**AA**	**SPM**
Human-APID	0.093	0.031	0.037	0.089	0.015	0.034	0.041	**0.097**
Human-BioGRID	**0.168**	0.091	0.129	0.152	0.032	0.089	0.111	0.135
Human-HINT	**0.141**	0.055	0.072	0.125	0.033	0.055	0.072	0.136
Human-IID	0.145	0.046	0.059	0.114	0.056	0.078	0.090	**0.167**
Yeast-BioGRID	0.217	0.162	**0.242**	0.207	0.030	0.108	0.149	0.173
Yeast-HINT	**0.235**	0.116	0.226	0.206	0.116	0.120	0.154	0.217

**Table 4:** Average runtimes (in minutes) with standard deviations (over 10 runs) on each of the human PPI networks studied. The choice of hyperparameter *t* for the quantum and classical walks was the same as was reported in the Results Section.

	Human				Yeast	
**Model**	**APID**	**BioGRID**	**IID**	**HINT**	**BioGRID**	**HINT**
QW-A	4.15 ± 0.05	1.05 ± 0.01	5.39 ± 0.14	4.52 ± 0.03	0.13 ± 0.00	0.39 ± 0.00
QW-L	4.69 ± 0.03	1.2 ± 0.01	6.03 ± 0.14	5.02 ± 0.03	0.14 ± 0.00	0.44 ± 0.00
CRW	3.23 ± 0.05	0.82 ± 0.02	4.43 ± 0.05	3.52 ± 0.08	0.05 ± 0.00	0.17 ± 0.00
L3	0.54 ± 0.05	0.1 ± 0.01	1.15 ± 0.04	0.55 ± 0.03	0.01 ± 0.00	0.1 ± 0.00
PA	0.23 ± 0.03	0.04 ± 0.01	0.33 ± 0.03	0.18 ± 0.03	0.01 ± 0.00	0.03 ± 0.00
CN	0.23 ± 0.04	0.04 ± 0.01	0.39 ± 0.04	0.21 ± 0.03	0.01 ± 0.00	0.03 ± 0.00
AA	0.27 ± 0.05	0.05 ± 0.01	0.41 ± 0.03	0.24 ± 0.03	0.01 ± 0.00	0.04 ± 0.00
SPM	27.28 ± 1.27	6.38 ± 0.03	29.68 ± 0.50	24.67 ± 0.11	0.84 ± 0.01	2.67 ± 0.01

**Table 5:** Area under the receiver operating characteristic curve for 10% edge removals, averaged over 10 runs. The highest AuROC for each network is shown in bold.

AuROC: 10% Removal								
**Network**	**QW-A**	**QW-L**	**CRW**	**L3**	**PA**	**CN**	**AA**	**SPM**
Human-APID	0.930	0.917	0.933	**0.936**	0.888	0.812	0.814	0.897
Human-BioGRID	0.932	0.928	0.935	**0.936**	0.888	0.877	0.879	0.901
Human-HINT	0.943	0.931	0.945	**0.947**	0.904	0.846	0.851	0.913
Human-IID	**0.945**	0.923	0.942	0.944	0.911	0.896	0.901	0.924
Yeast-BioGRID	0.914	0.911	**0.918**	0.917	0.838	0.873	0.878	0.876
Yeast-HINT	0.939	0.926	**0.946**	0.939	0.909	0.893	0.906	0.919

**Table 6:** Area under the receiver operating characteristic curve for 50% edge removals, averaged over 10 runs. The highest AuROC for each network is shown in bold.

AuROC: 50% Removal								
**Network**	**QW-A**	**QW-L**	**CRW**	**L3**	**PA**	**CN**	**AA**	**SPM**
Human-APID	0.910	0.900	**0.918**	0.908	0.883	0.717	0.717	0.870
Human-BioGRID	0.906	0.903	**0.910**	0.899	0.877	0.779	0.780	0.860
Human-HINT	0.924	0.915	**0.931**	0.925	0.898	0.760	0.762	0.879
Human-IID	0.930	0.915	0.934	**0.936**	0.909	0.838	0.841	0.898
Yeast-BioGRID	0.874	0.871	**0.877**	0.859	0.821	0.775	0.777	0.784
Yeast-HINT	0.922	0.910	**0.931**	0.926	0.904	0.833	0.845	0.890

## Data Availability

The datasets used in this study are available upon request from the corresponding authors.

## References

[1] Adamic L.A., Adar E. (2003). Friends and neighbors on the web. Soc. Netw..

[2] Murata T., Moriyasu S. Link prediction of social networks based on weighted proximity measures. Proceedings of the IEEE/WIC/ACM International Conference on Web Intelligence (WI’07).

[3] Leskovec J., Huttenlocher D., Kleinberg J. Predicting positive and negative links in online social networks. Proceedings of the 19th International Conference on World Wide Web.

[4] Kovács I.A., Luck K., Spirohn K., Wang Y., Pollis C., Schlabach S., Bian W., Kim D.K., Kishore N., Hao T. (2019). Network-based prediction of protein interactions. Nat. Commun..

[5] Liu W., Lü L. (2010). Link prediction based on local random walk. EPL Europhys. Lett..

[6] Kumar A., Singh S.S., Singh K., Biswas B. (2020). Link prediction techniques, applications, and performance: A survey. Phys. Stat. Mech. Its Appl..

[7] Martínez V., Berzal F., Talavera J.C.C. (2017). A Survey of Link Prediction in Complex Networks. ACM Comput. Surv..

[8] Zhou T. (2021). Progresses and challenges in link prediction. iScience.

[9] Che Y., Cheng W., Wang Y., Chen D. (2021). A Random Walk with Restart Model Based on Common Neighbors for Predicting the Clinical Drug Combinations on Coronary Heart Disease. J. Healthc. Eng..

[10] Zhou Y., Wu C., Tan L. (2021). Biased random walk with restart for link prediction with graph embedding method. Phys. A Stat. Mech. Its Appl..

[11] Brin S., Page L. (1998). The anatomy of a large-scale hypertextual web search engine. Comput. Netw. Isdn Syst..

[12] Das Sarma A., Molla A.R., Pandurangan G., Upfal E. Fast distributed pagerank computation. Proceedings of the International Conference on Distributed Computing and Networking.

[13] Fouss F., Pirotte A., Renders J.M., Saerens M. (2007). Random-walk computation of similarities between nodes of a graph with application to collaborative recommendation. IEEE Trans. Knowl. Data Eng..

[14] Pan J.Y., Yang H.J., Faloutsos C., Duygulu P. Automatic multimedia cross-modal correlation discovery. Proceedings of the 10th ACM SIGKDD International Conference on Knowledge Discovery and Data Mining.

[15] Tong H., Faloutsos C., Pan J.Y. Fast random walk with restart and its applications. Proceedings of the Sixth International Conference on Data Mining (ICDM’06).

[16] Farhi E., Gutmann S. (1998). Quantum computation and decision trees. Phys. Rev. A.

[17] Aharonov Y., Davidovich L., Zagury N. (1993). Quantum random walks. Phys. Rev. A.

[18] Kempe J. (2003). Quantum random walks: An introductory overview. Contemp. Phys..

[19] Venegas-Andraca S.E. (2012). Quantum walks: A comprehensive review. Quantum Inf. Process..

[20] Childs A.M. (2009). Universal computation by quantum walk. Phys. Rev. Lett..

[21] Mülken O., Blumen A. (2011). Continuous-time quantum walks: Models for coherent transport on complex networks. Phys. Rep..

[22] Qian J., Yang L., Yu Z., Liu S. (2017). Link prediction using discrete-time quantum walk. Teh. Vjesn..

[23] Moutinho J.A.P., Melo A., Coutinho B., Kovács I.A., Omar Y. (2023). Quantum link prediction in complex networks. Phys. Rev. A.

[24] Manouchehri K., Wang J. (2014). Physical Implementation of Quantum Walks.

[25] Young A.W., Eckner W.J., Schine N., Childs A.M., Kaufman A.M. (2022). Tweezer-programmable 2D quantum walks in a Hubbard-regime lattice. Science.

[26] Wang K., Shi Y., Xiao L., Wang J., Joglekar Y.N., Xue P. (2020). Experimental realization of continuous-time quantum walks on directed graphs and their application in PageRank. Optica.

[27] Tang H., Lin X.F., Feng Z., Chen J.Y., Gao J., Sun K., Wang C.Y., Lai P.C., Xu X.Y., Wang Y. (2018). Experimental two-dimensional quantum walk on a photonic chip. Sci. Adv..

[28] Peruzzo A., Lobino M., Matthews J.C.F., Matsuda N., Politi A., Poulios K., Zhou X.Q., Lahini Y., Ismail N., Wörhoff K. (2010). Quantum Walks of Correlated Photons. Science.

[29] Preiss P., Ma R., Tai E., Lukin A., Rispoli M., Zupancic P., Lahini Y., Islam R., Greiner M. (2015). Strongly correlated quantum walks in optical lattices. Science.

[30] Gong M., Wang S., Zha C., Chen M.C., Huang H.L., Wu Y., Zhu Q., Zhao Y., Li S., Guo S. (2021). Quantum walks on a programmable two-dimensional 62-qubit superconducting processor. Science.

[31] Yan Z., Zhang Y.R., Gong M., Wu Y., Zheng Y., Li S., Wang C., Liang F., Lin J., Xu Y. (2019). Strongly correlated quantum walks with a 12-qubit superconducting processor. Science.

[32] Loke T., Wang J.B. (2017). Efficient quantum circuits for continuous-time quantum walks on composite graphs. J. Phys. Math. Theor..

[33] Qiang X., Loke T., Montanaro A., Aungskunsiri K., Zhou X., O’Brien J.L., Wang J.B., Matthews J.C.F. (2016). Efficient quantum walk on a quantum processor. Nat. Commun..

[34] Vidal M., Cusick M.E., Barabási A.L. (2011). Interactome networks and human disease. Cell.

[35] Stelzl U., Worm U., Lalowski M., Haenig C., Brembeck F.H., Goehler H., Stroedicke M., Zenkner M., Schoenherr A., Koeppen S. (2005). A human protein–protein interaction network: A resource for annotating the proteome. Cell.

[36] Rolland T., Taşan M., Charloteaux B., Pevzner S.J., Zhong Q., Sahni N., Yi S., Lemmens I., Fontanillo C., Mosca R. (2014). A proteome-scale map of the human interactome network. Cell.

[37] Luck K., Kim D.K., Lambourne L., Spirohn K., Begg B.E., Bian W., Brignall R., Cafarelli T., Campos-Laborie F.J., Charloteaux B. (2020). A reference map of the human binary protein interactome. Nature.

[38] Yuen H.Y., Jansson J. Better Link Prediction for Protein-Protein Interaction Networks. Proceedings of the 2020 IEEE 20th International Conference on Bioinformatics and Bioengineering (BIBE).

[39] Yuen H.Y., Jansson J. (2023). Normalized L3-based link prediction in protein protein interaction networks. BMC Bioinform..

[40] Liben-Nowell D., Kleinberg J. (2007). The link-prediction problem for social networks. J. Am. Soc. Inf. Sci. Technol..

[41] Barabási A.L., Jeong H., Néda Z., Ravasz E., Schubert A., Vicsek T. (2002). Evolution of the social network of scientific collaborations. Phys. A Stat. Mech. Its Appl..

[42] Masuda N., Porter M.A., Lambiotte R. (2017). Random walks and diffusion on networks. Phys. Rep..

[43] Childs A.M., Farhi E., Gutmann S. (2002). An Example of the Difference Between Quantum and Classical Random Walks. Quantum Inf. Process..

[44] Thomas G. (2016). Wong, L.T.; Nahimov, N. Laplacian versus adjacency matrix in quantum walk search. Quantum Inf. Process..

[45] Childs A.M., Goldstone J. (2004). Spatial search by quantum walk. Phys. Rev. A.

[46] Stark C., Breitkreutz B.J., Reguly T., Boucher L., Breitkreutz A., Tyers M. (2006). BioGRID: A general repository for interaction datasets. Nucleic Acids Res..

[47] Das J., Yu H. (2012). HINT: High-quality protein interactomes and their applications in understanding human disease. BMC Syst. Biol..

[48] Alonso-López D., Campos-Laborie F.J., Gutiérrez M.A., Lambourne L., Calderwood M.A., Vidal M., De Las Rivas J. (2019). APID database: Redefining protein–protein interaction experimental evidences and binary interactomes. Database.

[49] Alonso-Lopez D., Gutiérrez M.A., Lopes K.P., Prieto C., Santamaría R., De Las Rivas J. (2016). APID interactomes: Providing proteome-based interactomes with controlled quality for multiple species and derived networks. Nucleic Acids Res..

[50] Kotlyar M., Pastrello C., Sheahan N., Jurisica I. (2016). Integrated interactions database: Tissue-specific view of the human and model organism interactomes. Nucleic Acids Res..

[51] Barabási A.L., Albert R. (1999). Emergence of scaling in random networks. Science.

[52] Hanley J.A., McNeil B.J. (1982). The meaning and use of the area under a receiver operating characteristic (ROC) curve. Radiology.

[53] Fawcett T. (2006). An introduction to ROC analysis. Pattern Recognit. Lett..

[54] Armengol E., Boixader D., Grimaldo F. (2015). Evaluating link prediction on large graphs. Artificial Intelligence Research and Development: Proceedings of the 18th International Conference of the Catalan Association for Artificial Intelligence.

[55] Saito T., Rehmsmeier M. (2015). The precision–recall plot is more informative than the ROC plot when evaluating binary classifiers on imbalanced datasets. PLoS ONE.

[56] Lü L., Pan L., Zhou T., Zhang Y.C., Stanley H. (2015). Toward link predictability of complex networks. Proc. Natl. Acad. Sci. USA.

[57] Zeng X., Liu L., Lü L., Zou Q. (2018). Prediction of potential disease-associated microRNAs using structural perturbation method. Bioinformatics.

[58] Holme P., Kim B.J. (2002). Growing scale-free networks with tunable clustering. Phys. Rev. E.

[59] Moler C., Van Loan C. (2003). Nineteen dubious ways to compute the exponential of a matrix, twenty-five years later. SIAM Rev..

[60] Paszke A., Gross S., Massa F., Lerer A., Bradbury J., Chanan G., Killeen T., Lin Z., Gimelshein N., Antiga L., Wallach H., Larochelle H., Beygelzimer A., d’Alché-Buc F., Fox E., Garnett R. (2019). PyTorch: An Imperative Style, High-Performance Deep Learning Library. Advances in Neural Information Processing Systems 32.

[61] Bader P., Blanes S., Casas F. (2019). Computing the matrix exponential with an optimized Taylor polynomial approximation. Mathematics.

[62] Strassen V. (1969). Gaussian Elimination is not Optimal. Numer. Math..

